# Multivariate projection method to investigate inflammation associated with secondary insults and outcome after human traumatic brain injury: a pilot study

**DOI:** 10.1186/s12974-016-0624-5

**Published:** 2016-06-21

**Authors:** Anna Teresa Mazzeo, Claudia Filippini, Rosalba Rosato, Vito Fanelli, Barbara Assenzio, Ian Piper, Timothy Howells, Ilaria Mastromauro, Maurizio Berardino, Alessandro Ducati, Luciana Mascia

**Affiliations:** Anesthesia and Intensive Care Unit, Department of Surgical Sciences, University of Torino, Torino, Italy; Department of Surgical Sciences, University of Torino, Torino, Italy; Department of Psychology, University of Torino, Torino, Italy; Department of Clinical Physics, Southern General Hospital, Glasgow, UK; Section of Neurosurgery, Department of Neuroscience, Uppsala University, Uppsala, Sweden; Anesthesia and Intensive Care Unit, AOU Citta’ della Salute e della Scienza di Torino, Presidio CTO, Torino, Italy; Neurosurgery Unit, Department of Neuroscience, University of Torino, Torino, Italy; Dipartimento di Scienze e Biotecnologie Medico Chirurgiche, Sapienza University of Rome, Rome, Italy

**Keywords:** Traumatic brain injury, Intracranial hypertension, Secondary insults, Neuroinflammation, Cytokines, Principal component analysis

## Abstract

**Background:**

Neuroinflammation has been proposed as a possible mechanism of brain damage after traumatic brain injury (TBI), but no consensus has been reached on the most relevant molecules. Furthermore, secondary insults occurring after TBI contribute to worsen neurological outcome in addition to the primary injury. We hypothesized that after TBI, a specific pattern of cytokines is related to secondary insults and outcome.

**Methods:**

A prospective observational clinical study was performed. Secondary insults by computerized multimodality monitoring system and systemic value of different cytokines were collected and analysed in the first week after intensive care unit admission. Neurological outcome was assessed at 6 months (GOSe). Multivariate projection technique was applied to analyse major sources of variation and collinearity within the cytokines dataset without a priori selecting potential relevant molecules.

**Results:**

Twenty-nine severe traumatic brain injury patients undergoing intracranial pressure monitoring were studied. In this pilot study, we demonstrated that after TBI, patients who suffered of prolonged and severe secondary brain damage are characterised by a specific pattern of cytokines. Patients evolving to brain death exhibited higher levels of inflammatory mediators compared to both patients with favorable and unfavorable neurological outcome at 6 months. Raised ICP and low cerebral perfusion pressure occurred in 21 % of good monitoring time. Furthermore, the principal components selected by multivariate projection technique were powerful predictors of neurological outcome.

**Conclusions:**

The multivariate projection method represents a valuable methodology to study neuroinflammation pattern occurring after secondary brain damage in severe TBI patients, overcoming multiple putative interactions between mediators and avoiding any subjective selection of relevant molecules.

## Background

Neuroinflammation is recognized as a key feature occurring after traumatic brain injury (TBI), and both localized and systemic inflammatory reactions have been proposed as potential mechanisms of damage or as putative beneficial responses to injury, depending on timing and severity [1–7]. Several cytokines, chemokines, and cell adhesion molecules have been identified in blood, cerebrospinal fluid (CSF), or brain microdialysate of patients with TBI with a highly variable profile in terms of peak and duration [5, 7–10].

After TBI, the injured brain is vulnerable to secondary damage which may be exacerbated by damaging events known as secondary insults contributing to worsen neurological outcome [11–15]. These harmful complications, occurring both in the prehospital phase and after intensive care unit (ICU) admission, include hypotension, hypoxia, high intracranial pressure, and nosocomial infection whose occurrence can be determined only if rigorously pursued after TBI [15–19]. The use of a minute by minute recording of physiological variables with a computerised multimodality monitoring system [18] can be applied to investigate basic mechanisms underlying secondary brain damage. These complications are indicative of secondary central nervous system (CNS) injury eventually occurring as a result of prolonged inflammation. Although several studies [1, 2, 4, 5, 7, 9, 20–23] have investigated the role of some given cytokines in the pathophysiology of TBI, there is no consensus on those who may serve as biomarkers of brain injury. Previous studies separately evaluated the relationship between selected cytokines and intracranial hypertension [22], hypoxemia [24], or the prognostic value of these mediators [25]. Among the main limitations of these studies on neuroinflammation, there are the multiple putative interactions between mediators which may vary together after TBI, and the limit of “a priori” selection of the potential relevant molecules [5, 8, 10]. Multivariate regression techniques are indeed limited to comparison of multivariate data in a large number of patients to prevent overfitting.

Multivariate projection methods, such as principal component analysis (PCA), and partial least squares (PLS), are data reduction techniques that allow the major sources of variation in a multi-dimensional dataset to be analysed avoiding the "a priori" selection of the potential relevant variables in a relatively small number of observations. PCA has been first proposed by Helmy et al. [9] in TBI patients to explore the pattern of production, time profile, and differing patterns of response of cytokines in brain and peripheral blood and then by Kumar et al. [5], who studied the prognostic value of a combination of CSF inflammatory molecules taking into account the variability across patients.

In the present study, we hypothesised that in patients with TBI, a specific pattern of cytokines and chemokines is related to secondary insults and may identify patients who die early because of brain herniation. To address the issue of mediators covariance we applied the multivariate projection techniques, including PCA and PLS analyses.

## Methods

### Patient population

The Institutional Review Board (Comitato Etico Interaziendale AOU Citta’ della Salute e della Scienza di Torino, Italy) approved the study protocol. At enrollment, patients were unconscious and unable to give consent, therefore the family was informed of the study, and consent was delayed until the patient was able to provide valid informed consent. Written permission for using collected data was then obtained from the patient or from the family (in case of death or if the patient remained incompetent to give consent). All patients with severe TBI consecutively admitted to the NeuroICU at the Azienda Ospedaliera Universitaria Citta’ della Salute e delle Scienza di Torino were prospectively recruited over a period of 3 years, according to the following inclusion criteria: age older than 18 years, severe head injury (Glasgow Coma Score (GCS) < 9) at ICU admission, placement of intracranial pressure (ICP) monitoring, and admission within 24 h after injury. Exclusion criteria were: both pupils fixed and dilated, history of immunosuppression, pregnancy, and lack of consent.

#### Clinical management

All the patients were sedated, intubated, mechanically ventilated, and managed according to the Brain Trauma Foundation Guidelines [26]. The GCS at the time of admission was recorded, and the Injury Severity Score (ISS) was used for the assessment of multiple injuries. Apache II score (Acute Physiology And Chronic Health Evaluation) was used for a quantification of the severity of illness and Marshall scale was used to classify head CT scan on admission. As part of the clinical management mean arterial pressure (MAP), intracranial pressure (ICP), cerebral perfusion pressure (CPP), oxygen saturation measured by pulse oximetry (SpO_2_), and temperature were continuously recorded.

#### Multimodality monitoring system for secondary insults detection

A computerized multimodality monitoring system was used for the collection of physiological parameters. A bedside laptop computer with specialized software displayed and saved one value per minute for each monitored physiological parameter. For the purpose of the study, data monitoring was started at the time of ICP placement and collected until ICP monitoring was discontinued for clinical reasons. Data were collected as part of a European network for collection and analysis of higher resolution data after TBI, the Brain Monitoring with Information Technology (BrainIT group) [27]. The Odin browser software was used for data analysis. Thresholds for secondary insults were derived from EUSIG [13]. The secondary insults analysed were raised ICP, low CPP, hypotension, hypoxia and pyrexia. Secondary insult thresholds, for grades 1, 2, and 3 were, respectively: ICP ≥ 20, ≥ 30, ≥ 40 mmHg for raised ICP insult; CPP ≤ 60, ≤50, ≤40 mmHg for low CPP insult; MAP ≤ 70, ≤ 55, ≤ 40 mmHg for hypotension insult; SaO2 ≤ 90, ≤85, ≤ 80 % for hypoxia insult; Temperature ≥ 38, ≥39, ≥ 40 °C for pyrexia insult. Each derangement had to be sustained for at least 5 min to be deemed a secondary insult, for 60 min in the case of pyrexia. The amount of secondary insult was calculated as the time spent within the insult threshold level divided by the good monitoring time (GMT) for that patient and presented as proportion of GMT [13, 18, 28, 29]. GMT is described as total monitoring time minus invalid monitoring or gaps in data collection for procedures, computerized tomography (CT) scan or system failures. All monitoring data were screened manually to disclose artifacts.

#### Neurological outcome

For evaluation of neurological outcome at 6 months the Glasgow Outcome Scale-extended (GOSe) was used, with a score ranging from 1 (death) to 8 (upper good recovery) [30, 31]. Dichotomization of outcome in favorable (GOSe 5–8) and unfavorable (GOSe1-4) was used; patients evolving to brain death in the early phase of ICU stay were identified as a separate group.

### Inflammatory mediators analysis

Blood samples for cytokine analysis were collected at the time of ICP placement (T0), 24 (T1), 48 (T2), and 72 h (T3) later. Samples were centrifuged for 10 min at 3000 RPM at 4 °C and plasma was then frozen at −80 °C until analysed. The cytokine analysis was performed with the Bioplex technology (BioRad Laboratories), which combines the principle of a sandwich immunoassay with fluorescent bead-based technology [32]. The Bioplex assay analyses 27 cytokines: Interleukin-1 beta (IL-1β), IL-1 receptor antagonist (IL-1ra), IL-2, IL-4, IL-5, IL-6, IL-7, IL-8, IL-9, IL-10, IL-12 subunit p70 (IL-12p70), IL-13, IL-15, IL-17, basic Fibroblast growth factor (basic FGF), eotaxin, granulocyte colony-stimulating factor (G-CSF), granulocyte-macrophage colony-stimulating factor (GM-CSF), interferon gamma (IFN-γ), interferon gamma-induced protein 10 (IP-10), monocyte chemoattractant protein 1 (MCP-1), macrophage inflammatory protein 1 alpha (MIP-1α), MIP-1β, Platelet-derived growth factor BB (PDGF-BB), Regulated upon Activation Normal T-cell Expressed (RANTES), Tumor necrosis factor alpha (TNF-α), Vascular endothelial growth factor (VEGF) and was carried out in 96-well microplates using the Bio-Plex Pro Human Cytokine 27-plex Assay kit following manufacture instruction (Code M50-0KCAF0Y, Bio-Rad Laboratories) at the Bioclarma-Research and Molecular Diagnostics, Torino, Italy. The intra-plate % coefficient of variance (CV) ranged from 1.11 to 9.96 %, while the inter-plate % CV ranged from 3 to 11 % for these assays.

All cytokine determinations on plasma samples were carried out in duplicate using Bio-Plex Manager software (vers 6.1). Soluble TNF-α receptors (TNF-RI and TNF-RII) was carried out using a solid-phase enzyme-linked immunosorbent assay method (ELISA) based on the quantitative immunometric sandwich enzyme immunoassay technique following the manifacture instruction (R&D Systems, Abingdon, UK). The intra-plate CV% was less than 4.8, while the inter-plate CV% was less than 5.1 for these assays. Plasma samples from 10 healthy volunteers were used as controls.

### Statistical analysis

In view of the inherent variation in absolute cytokine concentrations between patients, the median value for each cytokine was chosen for univariate analyses. Continuous data are presented as mean and standard deviation (SD) or median and interquartile range (IQR) depending on data distribution, while categorical data are presented as rate and proportion. Correlation between each cytokine and different secondary insults expressed as proportion of GMT was performed by linear regression analysis; differences among demographic data and cytokines level in the three outcome categories were tested using analysis of variance (ANOVA) or chi-squared test as appropriate and considered significant for *p* < 0.05. If ANOVA was significant, post-hoc analysis by Bonferroni was applied.

Multivariate projection is a data reduction technique that allows the major sources of variation in a multi-dimensional dataset to be analysed without introducing inherent bias. Principal component analysis (PCA) is used to identify principal components which account for the majority of the variation within the dataset. Number of principal components have been identified using Kaiser criteria (Eigenvalue >1).

Partial least squares (PLS) is a linear predictive model to maximize both the variation within the dataset and to the response variable. For multivariate projection techniques all cytokines values (T0, T1, T2 and T3) have been used after log-tranformation. The most significant cytokines in the PLS model were identified by the Variable Importance Projection (VIP). VIP is a measure of a variable’s importance in modeling both variation of cytokines, explained by each partial least squares factor, and variation of raised ICP insult. If a variable has a small coefficient and a small VIP (<0.8 -Wold’s criterion), then it is a candidate for deletion from the model.

In our study, PCA was applied to explore any intrinsic variation in the cytokine dataset while PLS was used to test the correlation between raised ICP (response variable) and cytokines. Finally, a multinomial generalized equation estimated (GEE) logistic regression model was applied to verify the predictive value of the principal components adjusted for clinical variables on neurological outcome. In order to correct for repeated measurements a robust sandwich standard error estimate was used. (SAS vers 9.3).

## Results

### Clinical data and occurrence of secondary insults

Twenty nine adult severe TBI patients (25 males and 4 females) were enrolled in the study. Demographic data of patients classified according to neurological outcome are presented in Table 1. Three patients presenting at admission with a GCS > 8 where included in the study as they suddenly deteriorated and met inclusion criteria. Difference in admission characteristics among favorable, unfavorable outcome at 6 months and brain-dead patients were not significant. Seventeen of the 29 enrolled patients suffered a polytrauma. The severity of trauma, assessed by ISS revealed a serious injury (ISS 9–15) in thre patients, a severe injury (ISS 16–24) in four patients, and a critical injury (ISS 25–75) in 22 patients. Five patients evolved to brain death within the first 3 days. GOSe at 6 months revealed: seven deaths including brain death (24 %), one persistent vegetative state (3 %), six upper or lower severe disability (21 %), eigth upper or lower moderate disability (28 %), seven lower or upper good recovery (24 %).

**Table 1:** Demographic data of the patient population in the three outcome groups (*n* = 29 Patients)

Variables	Favorable outcome 6M^*a*^	Unfavorable outcome 6M^*a*^	Brain death
	*n* = 15	*n* = 9	*n* = 5
Age (years), mean (SD)	35.9 (16.6)	40.8 (23.3)	42.2 (19.8)
Apache II, mean (SD)	14.1 (3.6)	15.6 (2.6)	16.6 (2.3)
GCS, median (IQR)	6 (3; 8)	4 (3; 5)	3 (3; 4)
GCSm, median (IQR)	4 (1; 4)	2 (1; 3)	1 (1; 1)
Marshall, median (IQR)	3 (2; 5)	3 (3; 5)	5 (4; 5)
Isolated TBI, *n* (%)	6 (40)	3 (33)	3 (60)
TBI in politrauma, *n* (%)	9 (60)	6 (67)	2 (40)
ISS, mean (SD)	27.5 (12.3)	28.3 (10.6)	26.2 (1.8)
AIS head, median (IQR)	4 (3; 4)	4 (3; 5)	5 (4; 5)
Focal injury, *n* (%)	4 (27)	3 (33)	3 (60)
Diffuse injury, *n* (%)	11 (73)	6 (67)	2 (40)
Main intracranial lesion, *n* (%)			
Epidural hematoma	2 (13)	0 (0)	1 (20)
Subdural hematoma	1 (7)	2 (22)	3 (60)
Traumatic subarachnoid hemorrhage	3 (20)	1 (11)	0 (0)
Contusions	7 (47)	2 (22)	1 (20)
Intracerebral mass lesion	1 (7)	2 (22)	0 (0)
Brain swelling	1 (7)	1 (11)	0 (0)
Diffuse axonal injury	0 (0)	1 (11)	0 (0)
Mechanism of injury, *n* (%)			
Fall	7 (47)	3 (33)	4 (80)
Motor vehicle collision	3 (20)	2 (22)	0 (0)
Motorcycle	1 (7)	3 (33)	1 (20)
Bicycle crash	2 (13)	0 (0)	0 (0)
Pedestrian	2 (13)	1 (11)	0 (0)

^a^At 6 months. *GCS* Glasgow coma scale, *GCSm* Glasgow coma scale, motor score, *TBI* traumatic brain injury, *ISS* injury severity score, *AIS* abbreviated injury scale

**Fig. 1:**
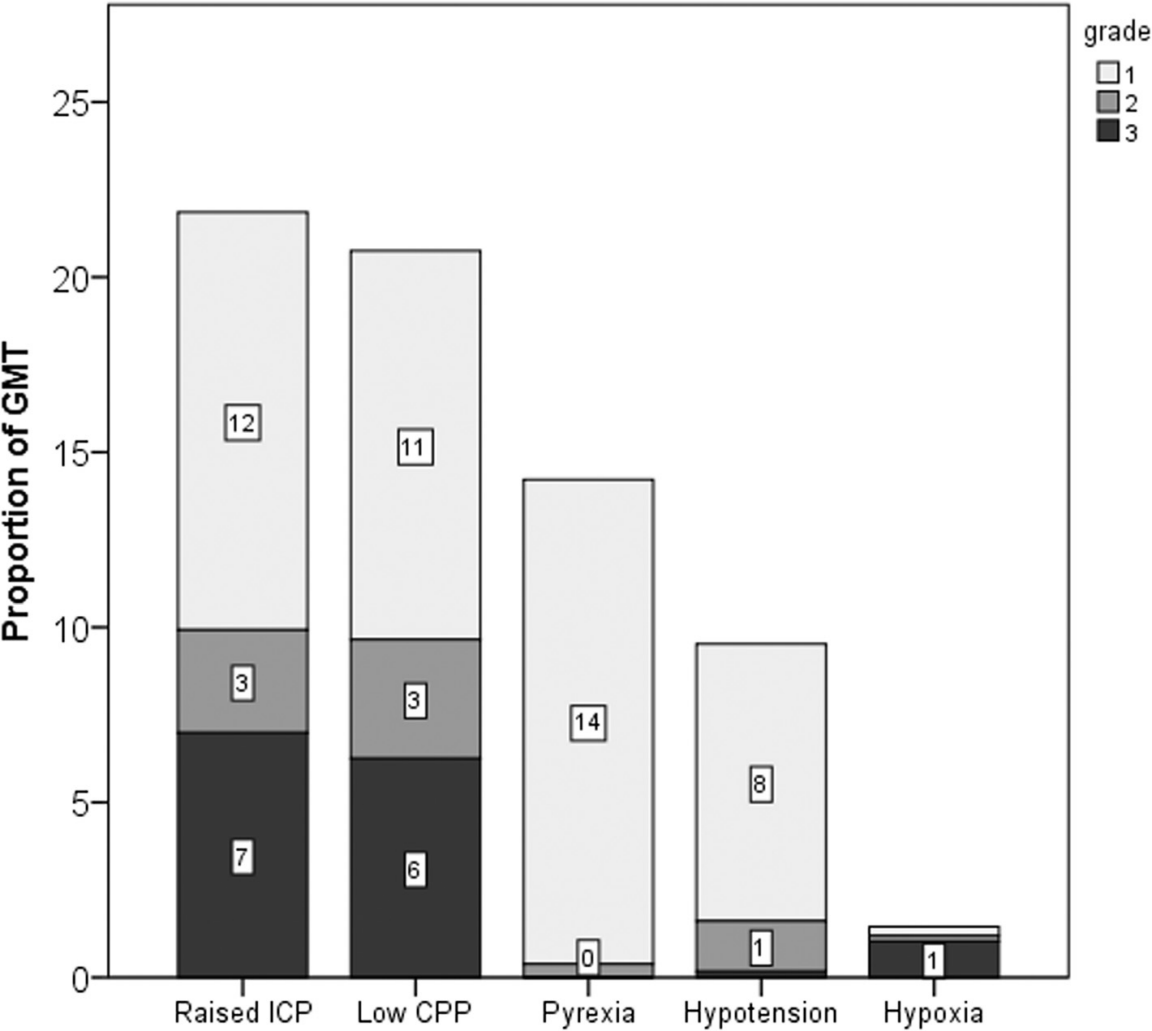
Occurrence of secondary insults, expressed as proportion of good monitoring time (GMT), for each insult category and each grade of severity. *ICP* intracranial pressure, *CPP* cerebral perfusion pressure. *Tags* inside *bars* indicate proportion of GMT for each insult grade

Median duration of GMT in the studied population was 7350 min (range 1191–13040). Occurrence of secondary insults during early phase of ICU stay, expressed as proportion of GMT for each grade, in each insult category is presented in Fig. 1. Raised ICP insult occurred in 21.9 % of GMT (CI 10.96; 32.75), low CPP insult in 21.1 % (CI 11.69; 30.59), pyrexia insult in 14.2 % (CI 9.23; 19.19), hypotension insult in 9.5 % (CI 4.61; 14.41) and hypoxia insult in 1.5 % (CI 0; 3.61). The majority of insults were of grade 1 and occurred in 11.9, 11.1, 13.8, and 7.9 % of GMT for raised ICP, low CPP, pyrexia and hypotension insult, respectively. Hypoxia insult in the early phase of ICU was rare but severe (grade 3). Median and range of secondary insults duration in the studied population were: raised ICP insult equal to 833 min (range 0–3513), low CPP insult equal to 904 (0–4095), pyrexia insult equal to 660 (0–3329), hypotension insult equal to 258 (0–2242), hypoxia insult equal to 0 (0–1637).

### Relationship between plasma cytokines level and secondary insults

A several-fold variation in the cytokine concentrations recovered was observed among patients. All cytokines were detectable in the analysed samples and increased from control levels while IL-2, MIP1α, IL-15, IL-17, and basic FGF were under detection limit in several patients. To deal with the repeated measures obtained from T0 to T3, we presented cytokines as median values over time. One patient died at T1 and had only two determinations.

**Fig. 2:**
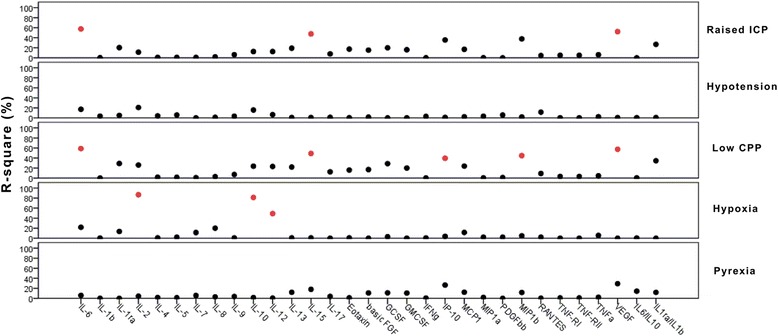
Linear regression analysis between median plasma levels of cytokines and secondary insults. The results are presented as percentage of R square between cytokines and secondary insults. *R* ^2^ > 0.5 and *p* < 0.01 are red colored. *ICP* intracranial pressure, *CPP* cerebral perfusion pressure

**Fig. 3:**
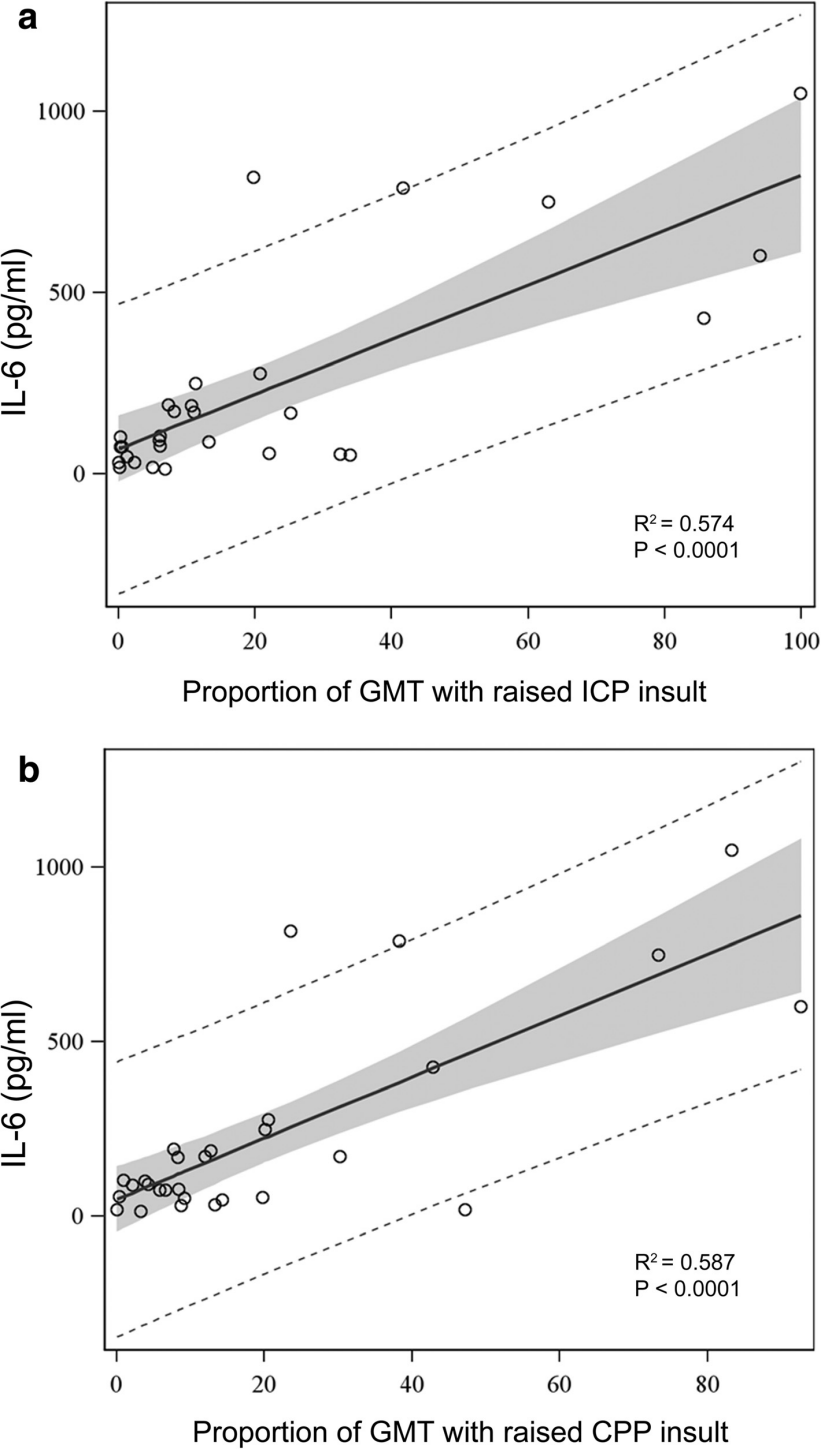
Linear regression analysis between median plasma levels of IL-6 and proportion of raised ICP **a** and low CPP **b** insults. Insults are expressed as proportion of good monitoring time (GMT). *Grey shadow* represents 95 % confidence limits. *Dotted lines* represent 95 % prediction limits. *ICP* intracranial pressure, *CPP* cerebral perfusion pressure

Il-6, IL-2, Il-10, IL-12, IL-15, VEGF, and MIP1β were the cytokines with the strongest correlation with secondary insults (*R*^2^ > 0.5 and *p* < 0.01). IL-6, IL-15 and VEGF were associated with ICP and low CPP insult, MIP1β with CPP insult, and IL-2, IL-10, and IL-12 with hypoxia insult (Fig. 2). IL-6 was the most important cytokine associated with raised ICP (*R*^2^ = 0.574, *p* < 0.0001) and low CPP insult (*R*^2^ = 0.587, *p* = 0.0001) (Fig. 3). All the correlations were confirmed when absolute numbers of minutes of insults were considered for analysis.

Correlations were found between plasma cytokine levels and demographic or severity scores at admission: age correlated with TNF-RI (*R*^2^ = 0.224, *p* = 0.009), TNF-RII (*R*^2^ = 0.270, *p* = 0.004) and VEGF (*R*^2^ = 0.129, *p* = 0.05); GCS motor score (GCSm) correlated with basicFGF (*R*^2^ = 0.146, *p* = 0.041), GCSF (*R*^2^ = 0.132, *p* = 0.05) and IP-10 (R^2^ = 0.167, *p* = 0.028); APACHE II score correlated with IP-10 (*R*^2^ = 0.154, *p* = 0.035), MIP1β (*R*^2^ = 0.134, *p* = 0.05), TNF-RII (*R*^2^ = 0.257, *p* = 0.005), and VEGF (*R*^2^ = 0.161, *p* = 0.031); Marshall scale correlated with IL-7 (*R*^2^ = 0.131, *p* = 0.05), IL-13 (*R*^2^ = 0.130, *p* = 0.05), IP-10 (*R*^2^ = 0.157, *p* = 0.033), and VEGF (*R*^2^ = 0.153, *p* = 0.036).

### Relationship between plasma cytokine levels and neurological outcome

**Fig. 4:**
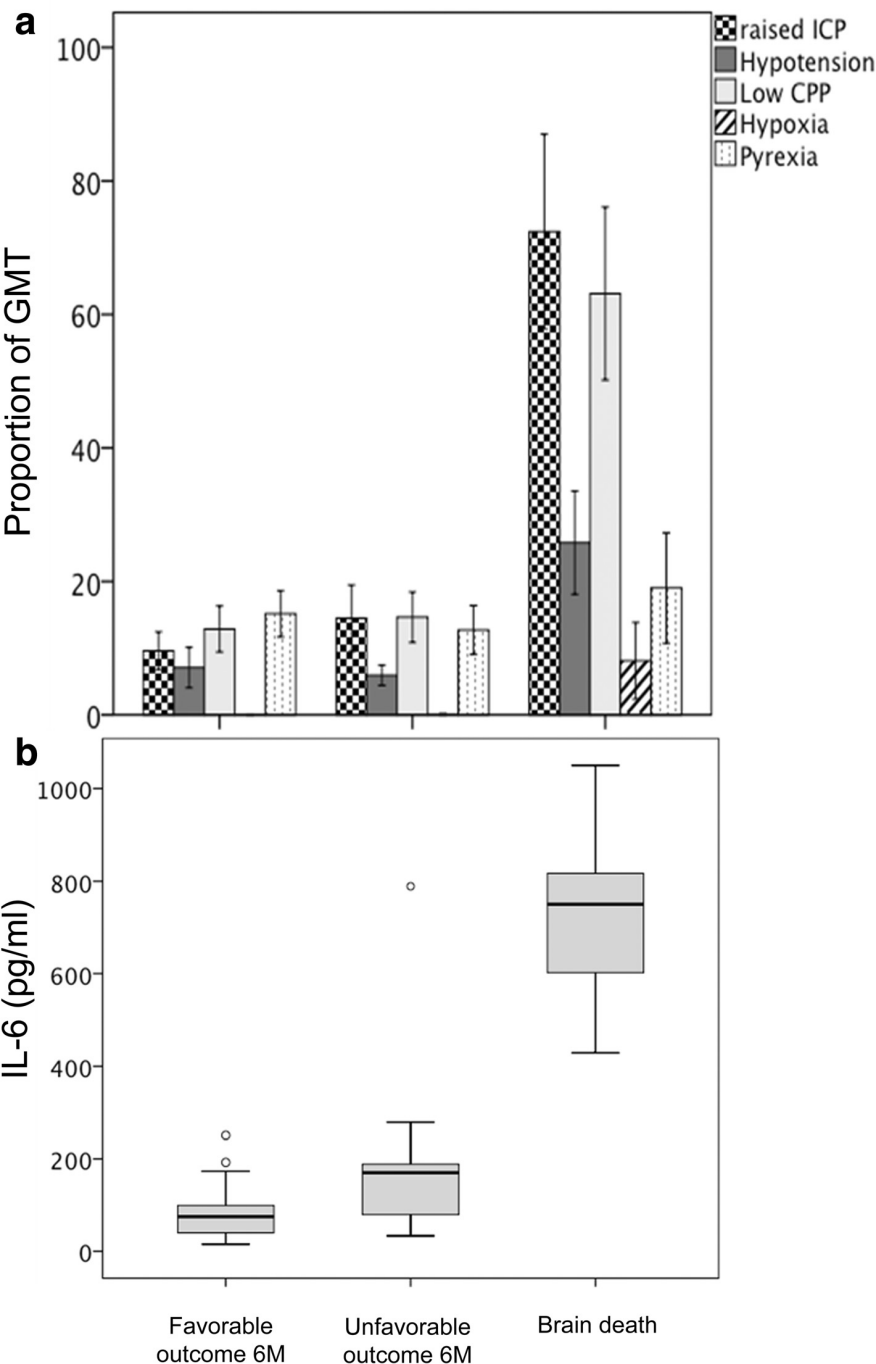
The incidence of secondary insults, expressed as proportion of good monitoring time (GMT) is described in each outcome group (**a**). Difference among groups was significant for raised ICP, hypotension, low CPP, and hypoxia insults (ANOVA, *p* < 0.01). Post-hoc analysis revealed that differences were significant between brain death and both favorable and unfavorable outcome (*p < 0.05). Data are expressed as mean and standard error. *ICP* intracranial pressure, *CPP* cerebral perfusion pressure. **b** Boxplots of IL-6 level in each outcome group. Difference among groups was significant (ANOVA, *p* < 0.001). Post-hoc analysis revealed that differences were significant between brain death and both favorable and unfavorable outcome (*p < 0.05). *Circles* represent outliers

Median plasma cytokine levels were significantly higher in patients evolving to brain death when compared to the other two groups (Table 2). Raised ICP, hypotension, low CPP, hypoxia insults (Fig. 4a) and IL-6 level (Fig. 4b) were significantly (*p* < 0.05) higher in patients early evolving to brain death compared to both patients with unfavorable and favorable outcome at 6 months.

**Table 2:** Median plasma level of inflammatory mediators in the 3 outcome groups

	Favorable outcome 6M^*a*^	Unfavorable outcome 6M^*a*^	Brain death	*p* value
IL-6	75.08 (31.81; 104.76)	169.95 (79.17; 188.3)	750 (602; 817)	*<0.0001*
IL-1β	0.66 (0.1; 1.05)	1.04 (0.6; 5.78)	1.32 (1.1; 1.97)	0.179
IL-1ra	66.92 (34.68; 226.97)	82.02 (70; 384.13)	532.66 (399; 2078.5)	0.078
IL-2	0.8 (0.8; 0.8)	0.8 (0.8; 0.8)	0.8 (0.8; 29.5)	*0.041*
IL-4	0.21 (0.01; 1.32)	0.6 (0.42; 5.31)	0.93 (0.8; 1.34)	0.304
IL-5	0.85 (0.13; 5.24)	1.14 (0.28; 15.36)	0.5 (0.29; 0.62)	0.415
IL-7	9.27 (1.38; 31.41)	45.4 (7.23; 141.17)	58.98 (16.25; 92.5)	0.215
IL-8	19.39 (3.99; 39.64)	60.29 (39; 104.58)	125.55 (37.02; 129)	*0.013*
IL-9	12.13 (2.35; 20.13)	32.91 (28; 46.86)	23.68 (20.5; 48.26)	*0.022*
IL-10	11.52 (1.57; 20.14)	20 (7.83; 57.02)	39.47 (12.37; 108.5)	*0.029*
IL-12	5.64 (0.81; 22.2)	11.82 (5.33; 42.48)	20.15 (17; 109.5)	*0.043*
IL-13	2.97 (0.62; 5.27)	7.4 (1.92; 11.27)	12.06 (5.78; 30)	*0.047*
IL-15	1.05 (0.06; 4.6)	0.06 (0.06; 6.16)	22.75 (3.7; 44.9)	*0.001*
IL-17	0.9 (0.9; 9.58)	49.06 (0.9; 75.33)	67.84 (28; 77.42)	0.162
Eotaxin	0.35 (0.35; 18.45)	19 (0.35; 29.31)	24.49 (10.5; 34.42)	0.094
basic FGF	3 (3; 24.45)	48.73 (3; 66.79)	49.61 (49.49; 131)	*0.053*
GCSF	50.95 (8.99; 138.65)	156.61 (107.5; 368.77)	483.17 (165; 491.86)	0.012
GMCSF	48 (2.29; 78.14)	39.18 (0.21; 91.61)	99.55 (83.84; 113)	0.084
IFNγ	4.15 (1.08; 25.73)	14.97 (11.99; 110.33)	25 (24.74; 29.08)	0.230
IP-10	111.06 (29; 256.19)	248.22 (215.09; 459.34)	2081.5 (528.43; 2232.08)	*<0.0001*
MCP1	162.16 (44.12; 307.83)	196.04 (77.78; 657.25)	690.82 (600; 2210)	*0.001*
MIP1α	1.5 (0.05; 2.33)	3.07 (1.2; 6.49)	3.71 (0.05; 11)	0.209
PDGFbb	141.57 (58.58; 229.67)	271.36 (195.11; 372.25)	104.64 (93.1; 210.46)	0.324
MIP1β	87.27 (55.98; 134.88)	123.05 (98; 133.01)	416 (275.42; 594)	*0.001*
RANTES	5193.61 (1952.35; 8553.06)	8903.57 (4098.28; 11442.69)	3570.05 (2309.71; 3831.31)	0.564
TNF-RI	1252.27 (522.38; 1568.18)	1320.45 (970.33; 1513.64)	1957 (1543.13; 2679)	0.394
TNF-RII	2190 (730; 4723.08)	4161.9 (3707.69; 5368.54)	5678 (4919; 6564.78)	*0.021*
TNFα	8 (5.58; 50.59)	5.5 (3.9; 93.05)	9.78 (8.12; 18.45)	0.262
VEGF	6.04 (0.08; 27.12)	29.79 (8.12; 42.22)	60.22 (34.28; 322)	*0.002*
IL6/IL10	57 (30.39; 324.61)	59.24 (39.89; 104.28)	277.07 (126.7; 1598.89)	0.765
IL1ra/IL1β	7.92 (3.66; 19.53)	8.9 (3.77; 22.4)	27.11 (5; 32.29)	0.064

^a^At 6 months. Values are median (IQR). *IQR* interquartile range. Significant correlations are reported in italics

### Multivariate projection method

**Fig. 5:**
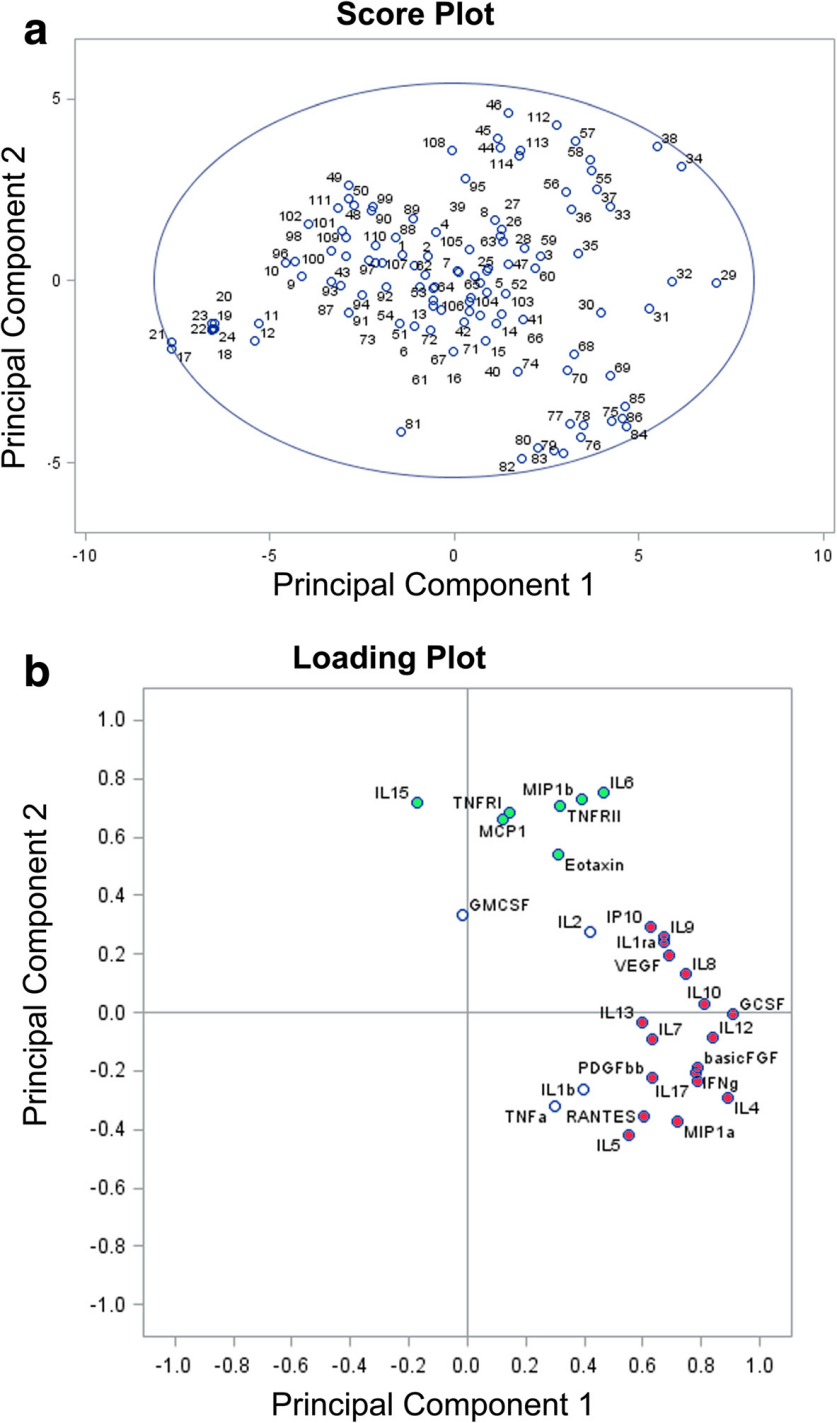
Panel **a**: Scores plot shows the scores on each principal component for each observation. The ellipse on the plot (Hotelling ellipse) is the 95 % CI for the model. **b** Loading plot shows the cytokines which load on the respective principal components. Cytokines which better explain Principal Components 1 and 2 are marked in *red* and *green*, respectively

PCA was performed to examine the presence of covariance in the cytokine dataset. According to Kaiser criteria, the first five PCs generated by the model explained 72 % of the cumulative variation within the dataset. Figure 5a shows the scores plot for each observation of PC1 and PC2 which explained 53 % of the cumulative variation within data set. The ellipse on the plot (Hotelling ellipse) represents the 95 % CI for the model and no outlier was detected. The loading plot (Fig. 5b) illustrates the relative contribution of each cytokine to the two PCs. The first component was strongly correlated with the following cytokines: GCSF, IL-4, IL-12, IL-10, basic FGF, IL-17, IFNγ, IL-8, MIP1α, VEGF, IL-1ra, IL-9, IL-7, PDGFbb, IP10, RANTES, IL-13, and IL-5. The second component was strongly correlated with IL-6, MIP1β, TNF-RII, Eotaxin, TNF-RI, MCP1, and IL-15. IL1β and TNFα were the cytokines which showed the highest coefficient of correlation within the third principal component; that explained the 8 % of variation within the dataset.

**Fig. 6:**
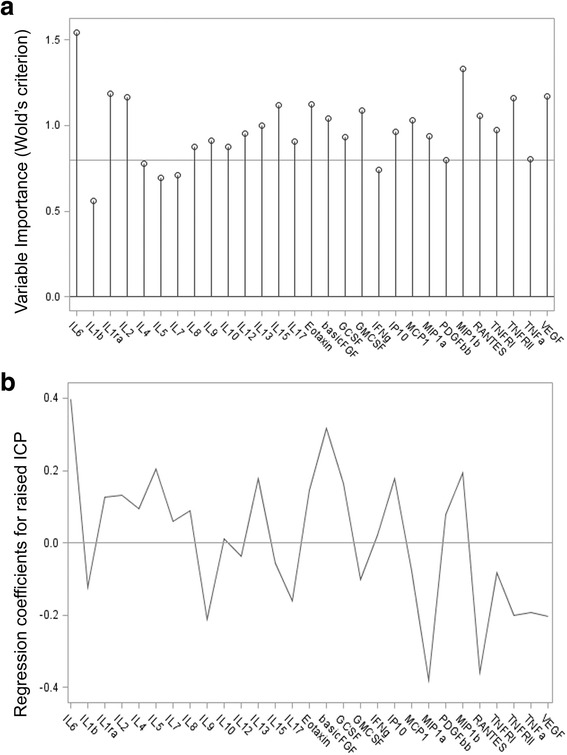
Panel **a** shows the variable importance projection (VIP) scores. A VIP score is a measure of a variable’s importance in modeling both variation of cytokines and variation of raised ICP insult. A value of 0.8 is generally considered to be a small VIP and a line is drawn on the plot at 0.8. Panel **b** is a regression coefficient profile indicating which cytokines better predict raised ICP insult. The regression coefficients represents the importance that each cytokine has in the prediction of raised ICP insult

Overall PLS analysis including the first five factors explained 63 % of cytokines variation and 53 % of raised ICP. According to the Wold’s criterion, in the PLS analysis IL-6, basic FGF, MIP1α, RANTES, IP10, MIP1β, explained most of the variation in the dataset and were the most powerful predictors for raised ICP (Fig. 6a and b).

To verify the predictive power of cytokine data on neurological outcome, the first two PCs, which explained most of the variability in the dataset, were entered in the multinomial logistic regression model together with demographic clinical variables (age, GCSm and Marshall score). GCSm was a powerful predictor discriminating patients with favorable outcome versus those who early died because of brain death. The first two PCs were significant predictors discriminating patients with favorable outcome versus both brain dead (OR = 1.91 [1.24; 2.94] and 4.64 [1.79; 12.05]) and unfavorable outcome (OR = 1.80 [1.34; 2.42] and 1.62 [1.02; 2.59], for PC1 and PC2 respectively, Table 3). The odds to evolve in brain death rather than favorable outcome increased 91 % for each unit increase in PC1.

**Table 3:** Multinomial logistic regression model of neurological outcome

Variables	OR [CI 95 %] vs favorable outcome	*p* value
Age		
Brain dead	0.96 [0.91; 1.02]	0.226
Unfavorable	0.98 [0.93; 1.04]	0.523
Marshall		
Brain dead	1.27 [0.28; 5.81]	0.762
Unfavorable	1.18 [0.46; 3.04]	0.730
GCSm		
Brain dead	0.37 [0.16; 0.85]	0.018
Unfavorable	0.98 [0.48; 2.01]	0.958
Principal component 1		
Brain dead	1.91 [1.24; 2.94]	0.003
Unfavorable	1.80 [1.34; 2.42]	<0.0001
Principal component 2		
Brain dead	4.64 [1.79; 12.05]	0.002
Unfavorable	1.62 [1.02; 2.59]	0.041

*GCSm* Glasgow Coma Scale, motor score. Unfavorable = unfavorable outcome at 6 months

Brain dead = brain dead patients

## Discussion

In this pilot study, we used a multivariate projection technique to identify distinct pattern of inflammatory response in TBI patients suffering of secondary insults. With this methodology, we demonstrated that patients who suffered from prolonged and severe secondary brain damage were characterized by a specific pattern of cytokines. Interestingly, in patients early evolving to brain death higher levels of inflammatory mediators were detected compared to both patients with long term favorable and unfavorable outcome. Keeping in mind the relatively small number of patients, the large number of cytokines and their putative statistical interactions, we applied the PCA to objectively identify the most relevant molecules in the early phase after TBI. The first 2 principal components explained 72 % of the variation within the dataset and were independently associated with poor neurological outcome. In the two components, the most relevant proinflammatory cytokines (GCSF, IL-6, IL-15), anti-inflammatory cytokines (IL-10, IL-1ra), chemokines, and growth factors (basicFGF, MIP1α, MIP1β, VEGF) were recognised by high coefficients. The PLS analysis identified IL-6, basic FGF, MIP1α, MIP1β, RANTES and IP10, as the main cytokines able to explain most of the variation in the dataset and to predict raised ICP.

Detrimental effects of secondary insults on TBI prognosis has been extensively investigated, but the exact mechanism leading to the exacerbation of brain damage remains unclear. Post-traumatic neuroinflammation, proposed as a potential mechanism of damage and repair, is characterised by glial activation, leukocyte recruitment and upregulation and secretion of mediators such as cytokines and chemotactic cytokines (chemokines) [3, 21, 22, 33, 34]. Inflammatory mediators have been measured in plasma, CSF and in brain microdialysate, suggesting a cerebral production of pro and anti-inflammatory cytokines [9]. In cases of brain injury complicated by multiple traumas, plasmatic levels of inflammatory mediators may reflect the effect of peripheral immune response [7]. Indeed recently Santarsieri et al. [6], demonstrated an association between CSF levels of inflammatory mediators in the first 6 days after injury and outcome modulated by cortisol levels. Recent experimental and clinical investigations data have also documented the role of microglia as source and target of inflammatory response [10, 35].

Cytokines produced by different CNS cells may have both beneficial and detrimental roles. Clear benefit can be achieved if the inflammation is controlled in a regulated manner and for a defined period of time; when sustained or excessive, however, inflammation is detrimental [36]. Unfortunately conflicting results have been reported on the role of different cytokines as repair mechanisms or exacerbation of the pathophysiology of brain trauma.

IL-6 is a multifunctional factor widely investigated in both experimental and clinical studies. Hergenroeder et al. [21] found that serum IL-6 levels within the first 24 h were significantly higher in patients who developed high ICP compared with patients with normal ICP. Minambres et al. [25] demonstrated that transcranial IL-6 gradient at admission correlated with poor prognosis at 6 months, and was significantly higher in patients evolving to brain death. Conversely Perez-Barcena et al. [37] did not identify a clear relationship between the temporal profile of IL-6 and ICP elevation, brain tissue oxygenation and the presence of brain swelling on CT scan. More recently, Kumar et al. using the group-based trajectory analysis demonstrated that patients with a high CSF IL-6 trajectory profile had worst outcome [5].

IL-1 cytokine family has been described as an important determinant of inflammation: IL-1α and IL-1β appear pro-inflammatory, while the endogenous IL-1ra appears anti-inflammatory. Thus elevation of IL-1ra/IL-1β ratio is seen as an anti-inflammatory indicator and in an elegant microdialysis study has been shown to be associated with better outcome [34, 38].

Chemokines contribution to secondary injury is mediated by accumulation of active leukocytes that perpetuates inflammation and neurotoxic cascades. In an observational clinical study the occurrence of hypoxemia after TBI was not associated with increased levels of IL-2, IL-6, and IL-10 but only with GM-CSF, S100 and myelin basic protein levels measured in CSF [24] while Stein et al. demonstrated that TNF-α and IL-8 were good predictors of both high ICP and low CPP recorded hourly by the chart [22, 33]. Recently Di Battista et al. [7] demonstrated the association between elevated IL-8, IL-10, TNF-α, MIP 1β, and MCP-1, hyperadrenergic state and poor outcome. The differences in study design, time window, and parameters analysed may account for much of the variability in these results.

Recently, the Wagner group [5] suggested that individual inflammatory markers may not be as informative of TBI pathology or predictive of outcome as an aggregated inflammatory score. In this perspective, they proposed a novel cytokine load score (CLS) and found a persistent inflammatory state with elevated serum IL-1β, IL-6, IL-8, IL-10, and TNFα levels over the first year post-injury, possibly as a result of a spillover effect from acute elevation after TBI; the proposed CLS was predictive of outcome at 6 and 12 months [5].

The main limitation in studies on neuroinflammation is indeed that authors mainly investigated the relationship between “a priori” selected cytokines and clinical variables, such as intracranial hypertension, hypoxemia or the prognostic value of these mediators. In these studies univariate correlations between a given mediator and a clinical outcome was applied to draw inferences regarding the biological action of the selected cytokine. From the pathophysiological point of view, this approach may be flawed because the primary injury is the common trigger for cytokines production and therefore is likely that these mediators will correlate with each other, particularly those cytokines that are directly antagonistic to one another at the same receptor.

The possible multiple collinearity among variables can be managed by data reduction methods but to prevent overfitting, large numbers of subjects in relation to the number of variables are required. Disentangling the profile and the inter-relationship between these mediators and avoiding the “a priori” selection of the potential relevant molecules is crucial to investigate their mechanistic role in the pathophysiology of TBI.

To overcome the problem of multiple variables with putative statistical interactions, multivariate projection techniques have been recently proposed. Helmy et al. used the PCA [8] to simplify multivariate data into few PCs that contains the main sources of variation within the dataset as a whole. These PCs are made up of a linear combination of the original variables, each of which contributes to a varying degree, termed the “loading”. The first PC is a linear combination of each of the original variables which incorporate the greatest source of variation within the dataset and will have a larger magnitude of coefficient than those contributing to a lesser degree. The second and subsequent PCs are further variables that explain the greatest sources of variation left over beyond the first PC. This analysis was used by Helmy et al. to demonstrate the different pattern of response in brain and peripheral blood of cytokines production and to demonstrate difference in cytokines profile after recombinant human IL-1ra administration in a phase II randomized control trial [10, 38].

In coherence with previous literature, in our study Il-6, IL-1ra, Il-8, IL-10, IL-15, MCP-1, MIP-1β, and IP10 were selected in the first two components of PCA as strong predictors of outcome confirming their role in the pathophysiology of brain injury. Among them IL-6, IP10, and MIP-1β remained in the predictive model for raised ICP. PCA analysis has also been recently used by Kumar et al. to explore the pattern of markers that contribute independently to variability in CSF inflammatory response. They found that Il-1β and TNFα provided limited contribution to variance suggesting that even if elevated, these markers had low discriminative capacity in inter-individual variability after TBI [5].

In our study, we collected data from ICU admission for the first 5 days and included also multiple trauma patients. It is therefore possible that an inflammatory response occurring within few hours from injury or after the first 5 days was missed in our database. Finally, the coexisting multiple trauma may have affected the extent of systemic inflammatory response in our patients and their impact on outcome.

A novel aspect of our study is related to the rigorous methodology that we used to record and collect secondary insults occurring early after ICU admission. In TBI patients following the initial event, secondary insults such as high ICP, low CPP, hypoxemia and pyrexia amplify the secondary damage and have been widely demonstrated to affect outcome. We resorted to the EUSIG scale proposed by Miller et al. [18] which is based on physiological thresholds to quantify occurrence and severity of secondary insults [13]. As part of the BrainIT group [27] in the present study, we collected high quality minute-by-minute physiological monitoring data using a standardized data collection equipment. Raised ICP and low CPP were the most frequent secondary insults occurring in almost 21 % of GMT. These results are consistent with literature, with intracranial hypertension occurring in 5–39 % of monitoring time depending on systems used to capture the insults and applied thresholds [28, 39, 40]. The strong predictive power of raised ICP on neurological outcome has been further confirmed by Güiza et al. [41]. Pyrexia occurred in 14 % of GMT, similarly to data reported with the same methodology [13, 42]. The occurrence of hypoxemia was rare (1.5 % of GMT) in our study, since patients were included after initial ICU stabilization. In the present study we separately analysed those patients who died because of brain herniation from those who had a poor outcome at 6 months. Those who evolved to brain death clearly represented the group with the worst secondary insults. Indeed intracranial hypertension was present for almost 70 % of the GMT. On the other hand, in the same time window, occurrence of secondary insults was not significantly different between patients with favorable and unfavorable outcome at 6 months. This result suggests that different factors such as late secondary insults [43] and non neurological complications may play a major role in determining long-term neurological outcome [44].

Due to the observational design of the study, we were not able to conclude if the inflammatory reaction should be considered as marker of severity or mediator of the secondary insults. However the multivariate projection method identified a specific pattern of inflammation overcoming putative interactions and avoiding any subjective selection of relevant molecules.

## Conclusions

With the use of multivariate projection method, we showed that patients with severe TBI were characterized by a specific pattern of inflammatory reaction associated with the occurrence of raised ICP. This pattern of cytokines was selected by the PCA as powerful predictor of both the conditions of unfavorable outcome and early brain death. Even if this preliminary analysis requires confirmation in larger studies, our results shed more light on the correlation between secondary insults, systemic inflammation and neurological outcome after TBI, and may help in the future to identify specific therapeutic targets that modulate inflammation.

## Abbreviations

Apache, Acute Physiology And Chronic Health Evaluation; Basic FGF, basic Fibroblast growth factor; BrainIT, Brain Monitoring with Information Technology; CLS, cytokine load score; CPP, cerebral perfusion pressure; CSF, cerebrospinal fluid; CT, computerized tomography; EUSIG, Edinburgh University secondary insult grading; GCS, Glasgow Coma Score; G-CSF, granulocyte colony-stimulating factor; GCSm, GCS motor score; GM-CSF, granulocyte-macrophage colony-stimulating factor; GMT, good monitoring time; GOSe, Glasgow Outcome Scale-extended; ICP, intracranial pressure; ICU, intensive care unit; IFN-γ, interferon gamma; IL-12p70, IL-12 subunit p70; IL-1ra,IL-1 receptor antagonist; IL-1β, Interleukin-1 beta; IP-10, interferon gamma-induced protein 10; IQR, interquartile range; ISS, Injury Severity Score; MAP, mean arterial pressure; MCP-1, monocyte chemoattractant protein 1; MIP-1α, macrophage inflammatory protein 1 alpha; PC, principal component; PCA, Principal component analysis; PDGF-BB, Platelet-derived growth factor BB; PLS, Partial Least Squares; RANTES, Regulated upon Activation Normal T-cell Expressed; SD, standard deviation; SpO_2_, oxygen saturation measured by pulse oximetry; TBI, traumatic brain injury; TCDB, Traumatic Coma Data Bank; TNF-RI, TNF-α receptors; TNF-α, Tumor necrosis factor alpha; VEGF, Vascular endothelial growth factor.
